# Investigation of Arc Stability in Wire Arc Additive Manufacturing of 2319 Aluminum Alloy

**DOI:** 10.3390/ma17215160

**Published:** 2024-10-23

**Authors:** Qiyu Gao, Feiyue Lyu, Leilei Wang, Xiaohong Zhan

**Affiliations:** College of Materials Science and Technology, Nanjing University of Aeronautics and Astronautics, Nanjing 211106, China; feiyue_lv@163.com (F.L.); wangll@nuaa.edu.cn (L.W.)

**Keywords:** 2319 aluminum alloy, wire arc additive manufacturing, arc stability, current and voltage signals, arc morphology

## Abstract

Wire Arc Additive Manufacturing (WAAM) technology, known for its low equipment and material costs, high material utilization, and high production efficiency, has found extensive applications in the fabrication of key components for the aerospace and aviation industries. The stability of the arc is crucial for the WAAM process as it directly affects the forming of the parts. In this study, the monitoring data of electrical signals and arc morphology during the WAAM process of 2319 aluminum alloy were investigated using a high-speed camera system and current/voltage acquisition system. By analyzing the current and voltage signals, as well as the arc imaging results, the influence of arc stability on the forming of the cladding layer was studied. The experimental results indicated that when both current and voltage exhibit regular periodic fluctuations, this manifests as a stable short-circuit droplet transition form, while sudden changes in these signals represent abnormal droplet transition forms. The adaptability of the process directly influenced the arc shape, thereby affecting the forming of the cladding layer. Under the process parameters of welding speed of 240 cm/min and wire feeding speed of 6.5 m/min, it was observed that the current signal exhibited a tight state and the variance of the arc width was minimized. This indicated that at a higher wire feeding speed, the droplet transfer frequency was increased. Under these process parameters, the arc output was more stable, resulting in a uniform metal coating.

## 1. Introduction

The material 2319 aluminum alloy is highly regarded in the aerospace industry due to its excellent thermal properties and high strength-to-weight ratio, making it ideal for the production of variable cross-section thin-walled components [1,2,3]. Traditional manufacturing methods of variable cross-section aluminum alloy thin-walled structures involving mechanical machining are associated with drawbacks such as material wastage and extended production timelines [4,5]. In contrast, Wire Arc Additive Manufacturing (WAAM) offers a compelling solution with its superior material utilization, adaptability for intricate component geometries, and enhanced manufacturing flexibility [6,7]. WAAM is an additive manufacturing process developed based on welding technology, where metal wire is heated and melted by an electric arc and then deposited layer by layer to construct a three-dimensional structure. Compared to laser and electron beam additive manufacturing technologies, WAAM offers high deposition rates, low equipment and material costs, large-scale manufacturing capabilities, and minimal post-processing requirements. Consequently, it demonstrates significant application advantages in the aerospace, shipbuilding, automotive, and energy industries. Among them, a controlled short arc process with wire retraction, namely, cold metal transition (CMT) by the Fronius company, has the advantages of a stable arc shape, reduced spatter, and low heat input. The WAAM system based on CMT technology is used to manufacture Al-Mg alloy walls, exhibiting good tensile and impact fracture properties. The results show that the Al-Mg alloy produced by WAAM-CMT has superior properties compared to traditional casting alloys [8]. Li et al. used WAAM-CMT to prepare Al-5.49Cu-0.4Mn-0.29Cd alloy in order to obtain a uniform microstructure and improve its mechanical properties. Based on the experimental results and theoretical analysis, the strengthening mechanism and fracture mechanism of the CMT-WAAM aluminum alloy were revealed [9]. It can be seen that this technology can better control shape quality and improve manufacturing efficiency [10].

Arc stability refers to the degree to which the arc maintains a stable formation during the welding process without experiencing phenomena such as arc interruption. A stable arc has the advantages of smooth droplet transition, small arc length variation, less short-circuit splashing, beautiful weld formation, and high welding quality. On the contrary, if the arc formation is unstable, causing severe splashing during droplet transition, poor weld formation, and easy formation of defects such as porosity or slag inclusion, the welding quality will be low [11,12]. The relationship between process parameters and arc morphology has become a major area of interest for researchers aiming to optimize WAAM process schemes. By establishing a comprehensive understanding of this correlation, it becomes possible to improve the overall performance and efficiency of WAAM processes.

By continuously monitoring key manufacturing parameters in WAAM, such as arc stability, current, voltage, temperature, and others, potential manufacturing defects can be promptly detected and corrected [13]. This ensures that the final products meet the expected levels of quality and performance. By monitoring the stability of the arc and making adjustments as necessary, process consistency is ensured, thereby reducing variations in the finished products [14]. This is crucial for meeting stringent quality standards and specifications. Therefore, process monitoring proves to be advantageous in gaining control over the forming quality in the additive manufacturing process, mitigating potential risks and reducing time, research, and development expenses [15,16].

Hauser et al. [17] investigated the WAAM of AW4043/AlSi5(wt.%) under various welding torch lead angles using high-speed imaging to observe welding phenomena, revealing that specific lead angles and interlayer temperatures caused fluctuation effects in the deposited structures, which could be detected at the arc frequency of the process. They concluded that a pushing WAAM process with a slightly tilted lead angle can mitigate these effects. Zhu et al. [18] monitored the arc current and voltage signals during the WAAM process to identify droplet transfer patterns. By calculating the probability density distribution, power spectrum distribution, and energy gradient of time-domain synchronous signals, the characteristics of metal droplet transfer and energy transfer were studied. Ke et al. [19] utilized high-speed imaging to capture the droplet transfer process, which was then employed to establish a periodic droplet transfer model. Doumanidis et al. [20] investigated the influence of welding speed and wire feed rate on the geometric dimensions of molten droplets in the WAAM process. By varying the process parameters, they were able to control the size of the molten droplets and achieve precise control over the macroscopic morphology of the WAAM components.

In summary, many researchers and experts have found that process parameters have a significant impact on arc stability [21], manifested in changes in the geometry of the deposition layer [14], arc morphology [22], and voltage/current signals [23]. However, the basic physical factors that affect arc stability and their mechanisms of action need to be further studied. Therefore, this study utilized a high-speed camera system and current/voltage acquisition system to collect monitoring data on electrical signals and arc morphology and systematically analyzed the current and voltage signals as well as arc imaging results. The aim was to investigate the effects of welding speed and wire feeding speed on surface forming quality, electrical signal changes, droplet transition behavior, and arc morphology during the WAAM process; screen the influencing factors of arc stability; optimize parameters; and achieve stable and high-quality cladding layer formation.

## 2. Materials and Methods

**Figure 1:**
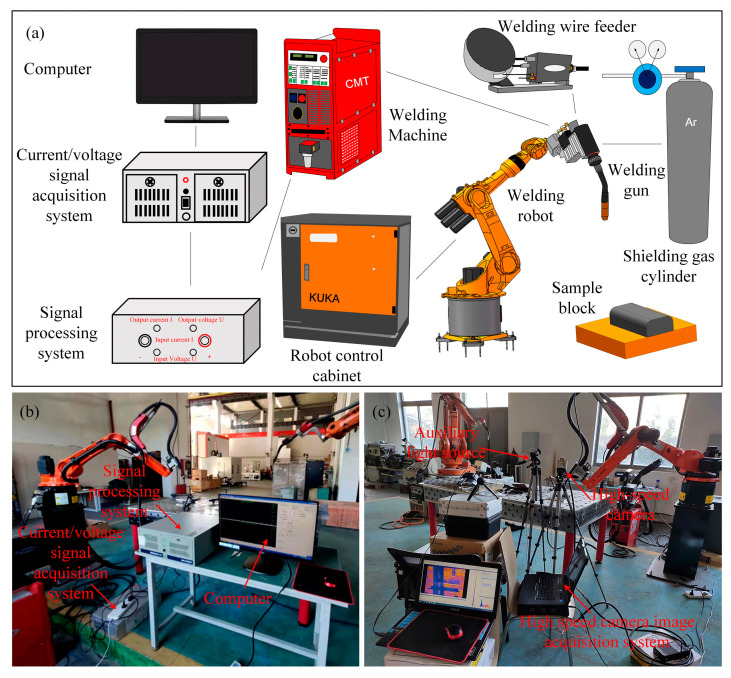
Types of equipment of WAAM experiments: (**a**) model diagram; (**b**) signal acquisition system; (**c**) high-speed camera image acquisition system.

The WAAM equipment and signal acquisition system are shown in Figure 1, including a Trans Plus Synergic 4000 CMT welding machine, KUKA KR10R 1420 C4 robot, VR1500 4R/F++ROBOTER wire feeding mechanism, current/voltage signal acquisition system, and AcutEye high-speed camera system (developed by Hunan Kotianjian Optoelectronics Technology Co., Ltd. (Changsha, China), equipped with a German Optronis high-speed camera, matching laptop industrial computer, and AcutEye software v4.0). The high-speed camera was positioned approximately 0.5 m away from the workpiece, angled at 45° relative to the horizontal worktable. Additionally, a filter lens was mounted on the front of the high-speed camera, which leveraged the light source emitted by the laser emitter to capture the arc morphology during the printing process. The electrical signal monitoring system used consisted of a current/voltage signal acquisition system, a signal processing system, and a computer. The current/voltage signal acquisition system was connected to the CMT welding machine to dynamically collect the current and voltage values output by the welding machine in real time. The acquisition system included a sampling amplification circuit and a data acquisition card. This data acquisition card had four differential input analog channels, with the core being a 12-bit A/D converter ADS7800, and a single acquisition time of 5000 ms. The analog channel of the acquisition card was sequentially connected to the arc voltage and welding current signals. The collected signals were transmitted through cables to the signal processing system for waveform analysis and, finally, the waveform display software in the computer was used to monitor the real-time changes in current and voltage during the arc additive process. This signal processing system could perform synchronous sampling and waveform analysis on multi-channel signals and calculate and process time-domain parameters such as the average value, effective value, and peak value of the signal. In addition, the signal processing system could achieve functions such as wavelet denoising, wavelet analysis, power spectrum estimation, and optimal feature subset extraction of signal sources. The electrical signals collected during the arc feeding process were current and arc voltage signals, where the voltage signal was usually proportional to the arc length. The formation of the arc and the transition of the droplet caused periodic changes in the arc length and resistance in the feeding circuit, thereby affecting the periodic changes in current/voltage in the feeding circuit. Therefore, when there were phenomena such as arc breaking and extinguishing during the additive process, the fluctuation of arc voltage and current could indirectly reflect the stability of the arc. The chemical compositions of AA2319 wire and AA2219 substrate are presented in Table 1, which were obtained based on Inductively Coupled Plasma Emission Spectrometer (ICP) experimental results.

The process parameters (welding machine settings) for WAAM are shown in Table 2. This method adopted a synergic mode to adjust the wire feeding speed, arc voltage, and current. That is, by selecting the wire feeding speed, the welding machine automatically set the arc voltage and current accordingly. The welding gun was perpendicular to the substrate, the dry elongation of the welding wire was set to 10 mm, and the welding path of the welding gun was S-shaped. In the CMT WAAM process, the wire underwent periodic retraction, facilitating the detachment of the droplet from the wire. This ensured that the droplet transfer occurred with an extremely low current value, enabling the continuous alternation between “cold” and “hot” states during the additive process. In addition, to prevent impurities from entering the molten pool during the WAAM process, resulting in material oxidation or increased porosity defects, argon gas with a purity of 99.99% [24,25] was used for local protection, and the shielding gas nozzle diameter was 30 mm. This gas was output coaxial through the welding gun, with a gas flow rate of 23 L/min. The target size of the WAAM test block was 130 mm × 20 mm × 10 mm (length × width × height). The test block was printed in 8 layers and a single weld bead with one layer, with an average layer height of 1.5 mm.

## 3. Results and Discussion

### 3.1. Macroscopic Morphology

**Figure 2:**
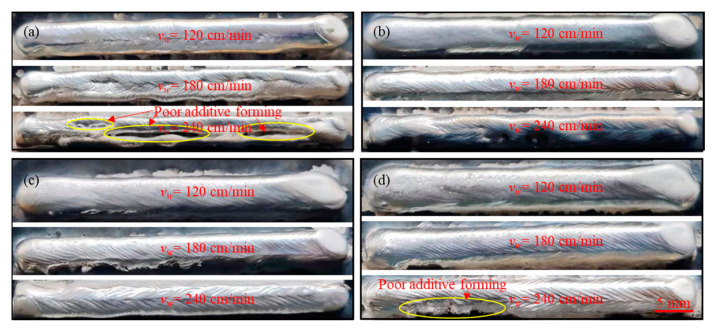
The influence of welding speed changes on AA2319 WAAM forming quality: (**a**) wire feeding speed 5.5 m/min; (**b**) wire feeding speed 6.0 m/min; (**c**) wire feeding speed 6.5 m/min, (**d**) wire feeding speed 7.0 m/min.

Figure 2 shows the surface forming quality of the WAAM samples with a welding speed of 120~240 cm/min and wire feeding rates of 5.5 m/min, 6.0 m/min, 6.5 m/min, and 7.0 m/min, respectively. As shown in Figure 2a, when the wire feeding rate was 5.5 m/min, the formation of the upper surface of the cladding layer was poor. When the welding speed was 120 cm/min, the ripples on the surface of the cladding layer were irregular and the ripples in the middle of the weld bead were pronounced but lacked continuity. When the welding speed was 180 cm/min, the surface of the cladding layer was non-uniform, with intermittent ripples present in the middle area of the cladding layer surface. When the welding speed was 240 cm/min, the ripples on the surface of the cladding layer were highly irregular, and serious intermittent ripples were present in the middle area of the cladding layer surface.

As shown in Figure 2b, at a welding speed of 120 cm/min, the continuity of the ripples on the upper surface of the cladding layer was enhanced, with no evident discontinuities or irregularities midway. However, the width of the top surface of the cladding layer increased compared to the condition with a wire feed speed of 5.5 m/min. At a welding speed of 180 cm/min, the surface of the cladding layer exhibited smooth and aesthetically pleasing welding ripples, indicating optimal heat input under these process parameters. This resulted in uniform metal deposition within the cladding layer. At a welding speed of 240 cm/min, the surface of the cladding layer became non-uniform, with fewer porosities and solidified droplets observed in the central region of the cladding layer.

As illustrated in Figure 2c, with a wire feed speed of 6.5 m/min, the surface of the cladding layer appeared smooth and glossy, and the consistency of the weld bead ripple was well maintained. When the welding speed was set to 120 cm/min, the width of the additive cladding layer increased further. However, at a welding speed of 180 cm/min, a slight collapse was observed at the right end of the cladding layer in the arc termination zone, likely due to a low arc stopping current or a short arc stopping time. A welding speed of 240 cm/min resulted in relatively high side roughness on the cladding layer.

As depicted in Figure 2d, with a wire feed speed of 7.0 m/min, the upper surface of the cladding layer remained smooth and flat, and the cladding layer width increased further. This was attributed to the higher wire feed speed resulting in an increased matching current and voltage of the welding machine, which, in turn, increased the heat input during the arc deposition process. This elevated heat input promoted extensive spreading of the molten metal and led to a wider cladding layer upon rapid solidification. At a welding speed of 120 cm/min, a noticeable collapse was observed at the start/stop positions of the arc. When the welding speed was 180 cm/min, a slight collapse occurred at the arc termination point on the right end, which could be attributed to a low arc stopping current or a short arc stopping time. At a welding speed of 240 cm/min, the cladding layer exhibited relatively high side surface roughness.

### 3.2. Changes in Electrical Signals

**Figure 3:**
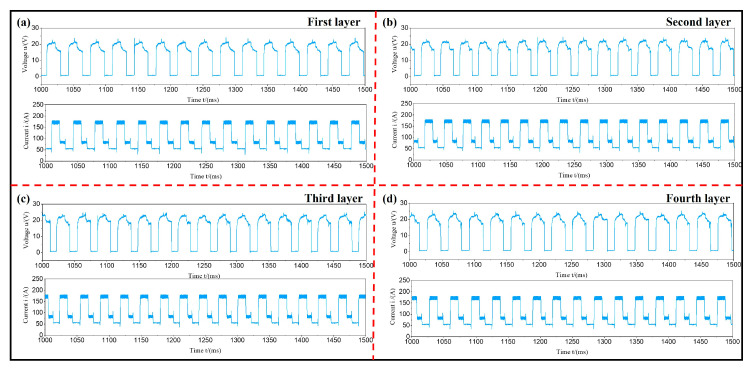
The variation of voltage/current signal with time at different layers at a welding speed of 120 cm/min and a wire feeding rate of 5.5 m/min: (**a**) first layer; (**b**) second layer; (**c**) third layer; (**d**) fourth layer.

The changes in voltage/current signals with time at different layers at a welding speed of 120 cm/min and a wire feeding rate of 5.5 m/min are shown in Figure 3. The results of the 1000~15,000 ms WAAM process indicated that there were no abnormalities in the current and voltage signals. During the WAAM process, the wire was sent out to make contact with the substrate and start arc striking. At this point, the arc melted the substrate and formed a molten pool. Next, the wire melted and formed droplets, which quickly transitioned into the molten pool. At this point, the corresponding electrical signals during the addition process underwent periodic changes.

**Figure 4:**
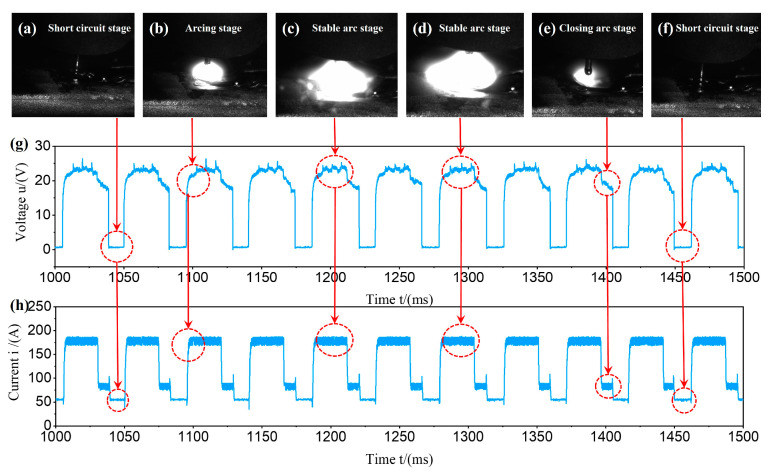
The relationship between the arc droplet transfer process and the current/voltage waveform in WAAM: (**a**) short circuit stage; (**b**) Arcing stage; (**c**) stable arc stage; (**d**) stable arc stage; (**e**) closing arc stage; (**f**) short circuit stage; (**g**) variation of voltage signal with time; (**h**) variation of current signal with time.

Figure 4 shows the relationship between the arc droplet transfer process and the current/voltage waveform during the WAAM process. As shown in Figure 4a, the droplet underwent a short-circuit transition and the current value rapidly dropped below 60 A, while the voltage value dropped sharply to 0.5–0.8 V. Subsequently, the wire feeding speed reversed, the wire was pulled back, and the current and voltage were almost zero. As shown in Figure 4b, the current/voltage value immediately increased and the arc was reignited. As shown in Figure 4c,d, within 40 ms, the current/voltage values remained basically unchanged, that is, this was the peak time of the current. The end of the wire continued to heat up, causing the droplet to grow. Meanwhile, under the action of the electromagnetic contraction force, the root of the droplet shrank. As shown in Figure 4e, during the arc closing phase, the current and voltage values slightly decreased. The welding wire was gradually fed downward and the arc width gradually decreased. When the welding wire came into contact with the molten pool, the droplet was once again sent into the molten pool through a short-circuit transition.

**Figure 5:**
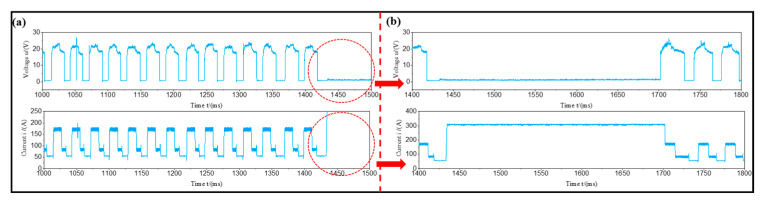
The variation of voltage/current signal with time at the third layer at a welding speed of 240 cm/min and a wire feeding rate of 5.5 m/min: (**a**) at 1000 ms~1500 ms; (**b**) at 1400 ms~1800 ms.

By analyzing the current/voltage signal waveform of the intercepted 1000~15,000 ms WAAM process, it was found that there were abnormal phenomena in the current/pressure signal of the third layer. The current and voltage waveforms were continuously monitored up until 1800 ms. Through observation, it was found that the voltage of the third layer was 0 V and the current reached 300 A. This was because the wire was in contact with the substrate at this time, and the arc voltage was 0 V. However, the current continued to reach the substrate through the wire, generating an instantaneous current of up to 300 A, as shown in Figure 5. The reason for this phenomenon was that during the arc feeding process, the wire continuously extended and retracted, leading to periodic fluctuations in current and voltage signals. The wire did not always interact with the workpiece. When the two were tightly connected and could not be separated, this could lead to wire-sticking behavior. This led to a tight connection between the cathode and anode without generating a potential difference. Therefore, if the arc voltage value was 0 V and the resistance value of the wire was low, the internal current value immediately increased to 300 A. Consequently, in this process combination, the welding speed was higher and the wire feeding speed was lower, resulting in extremely low heat input values in the addition process. The wire could not be transported by jet but could only enter the molten pool through short-circuit transportation. At the same time, due to low heat input, the wire could not fully melt, resulting in poor spreading of the cladding layer and smaller overlapping areas between the weld beads. This led to more voids in the middle of the cladding layer.

### 3.3. Droplet Transfer

**Figure 6:**
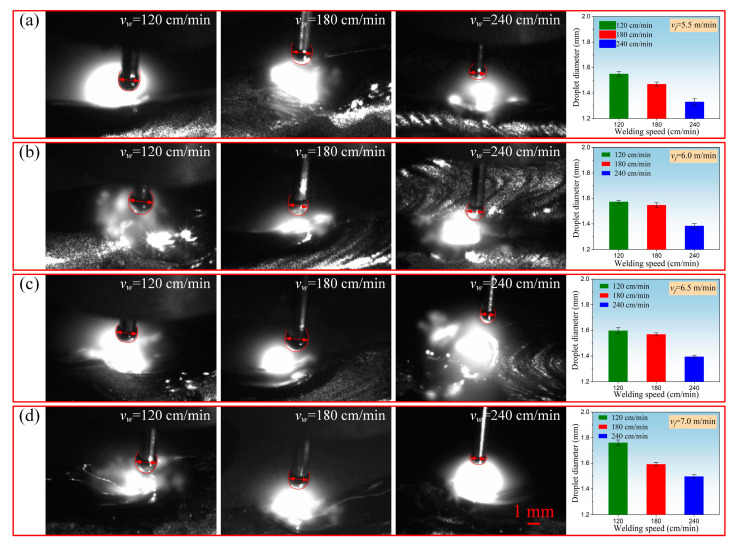
Droplet transfer behavior during arc additive process: (**a**) wire feeding speed 5.5 m/min; (**b**) wire feeding speed 6.0 m/min; (**c**) wire feeding speed 6.5 m/min; (**d**) wire feeding speed 7.0 m/min.

Real-time monitoring of the droplet morphology and droplet drop process during the arc addition process of 2319 aluminum alloy was carried out through high-speed cameras. In the closing arc stage, when the maximum droplet was formed at the tip of the welding wire, the maximum diameter size of the droplet was measured three times in each experiment, and the average value was taken to analyze the influence of welding speed and wire feeding speed on the shape and size of the droplet, as shown in Figure 6.

During the WAAM process, the droplet transfer remained in the form of short-circuit transfer under different process parameters, and the droplet contacted the molten pool before it grew, forming a short circuit. The shape of the droplet remained basically circular or nearly circular, and the overall width of the droplet was distributed from 1.3 mm to 2.2 mm. Due to the impact of arc recoil force and the surface tension of the wire, some of the droplet sizes decreased in the vertical direction and increased in the horizontal direction, forming an elliptical droplet. Therefore, the overall width and size distribution of the droplet were relatively wide, and the droplet size changed significantly under a single parameter. In addition, from Figure 6, we can see that the droplet size under all parameters was greater than the wire size. This was because there was surface tension at the contact surface between the wire and the droplet, which was related to the material, wire diameter, gas medium, and temperature. The direction of surface tension was opposite to gravity and had a certain limiting effect on the drop of molten droplets. Only when the droplet continued to grow until the size of the droplet reached a certain level, allowing the gravity of the droplet to overcome resistance, could it enter the molten pool.

As depicted in Figure 6, there was a negative correlation between the droplet size and welding speed. When the wire feeding speed remained constant, increasing the welding speed resulted in smaller droplet sizes. This was because a faster welding speed led to faster movement of the welding gun, resulting in reduced heat input to the molten pool per unit time and area. Consequently, there was a decrease in the amount of metal filling entering the molten pool. As the number of printing layers increased, the distance between the welding gun and the upper surface cladding layer also increased, causing the arc length to increase. This led to noticeable heat dissipation in the arc column area. Simultaneously, as the arc heat and resistance heat decreased, the heat that promoted droplet growth also decreased. Consequently, the melting amount of the wire gradually reduced, resulting in smaller droplet sizes during the formation process.

### 3.4. Evolution of Arc Morphology

**Figure 7:**
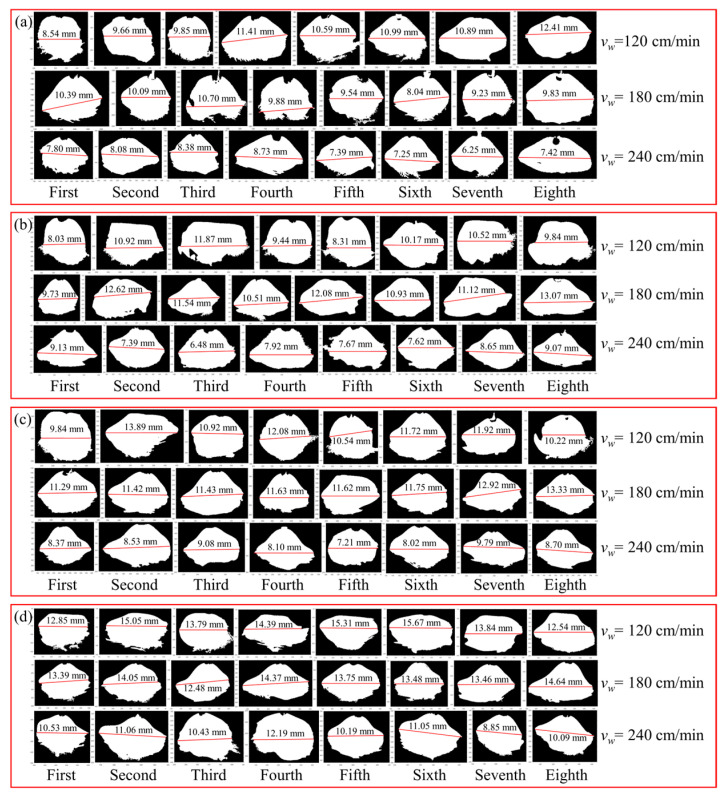
Effect of welding speed variation on arc morphology at different layer heights: (**a**) wire feeding speed 5.5 m/min; (**b**) wire feeding speed 6.0 m/min; (**c**) wire feeding speed 6.5 m/min; (**d**) wire feeding speed 7.0 m/min.

Observation and measurements were conducted on the region with the maximum arc expansion within a certain period of time, as shown in Figure 7. During the WAAM process, the arc morphology was close to a bell shape. When the wire feeding speed was low, the welding gun lifted by 1.5 mm per layer, while the amount of cladding per layer was less than 1.5 mm, resulting in an increasing distance between the welding gun and the upper surface of the cladding layer. The arc first diverged and then converged due to the electromagnetic contraction force, ultimately forming a bell-shaped arc. When the distance between the welding gun and the substrate was very long, the arc was elongated and the arc voltage increased. In order to maintain a stable voltage value, the welding machine slightly increased the current value, resulting in an increase in the amount of metal melted per unit time and an increase in the amount of cladding per layer. Therefore, the distance between the welding gun and the substrate returned to the original value, and the arc was sprayed radially, with a larger arc width.

**Figure 8:**
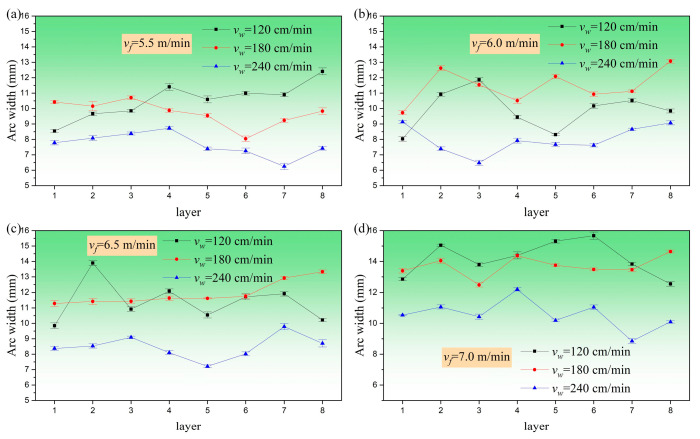
Curve plot of the effect of welding speed changes on arc morphology at different layer heights: (**a**) wire feeding speed 5.5 m/min; (**b**) wire feeding speed 6.0 m/min; (**c**) wire feeding speed 6.5 m/min; (**d**) wire feeding speed 7.0 m/min.

The maximum width of the extracted arc contour was measured using Image Pro Plus software v6.0, with three measurements taken each time. The average value of the results is shown in Figure 8, which illustrates the impact of different layer height/welding speed variations on the arc. When the given wire feeding speed was consistent, the arc width showed a decreasing trend as the welding speed increased from 180 cm/min to 240 cm/min. This was because as the welding speed increased and the matching current and voltage values of the welding machine were constant, the heat input per unit time in a fixed size area decreased and the mass of droplets entering the molten pool per unit time decreased. When the single-layer cladding amount was less than the lifting amount of each layer of the welding gun, the distance between the welding gun and the upper surface of the cladding layer gradually increased, the arc length increased, and the width decreased. The end of the arc converged due to the electromagnetic contraction force, ultimately forming a bell-shaped arc.

**Figure 9:**
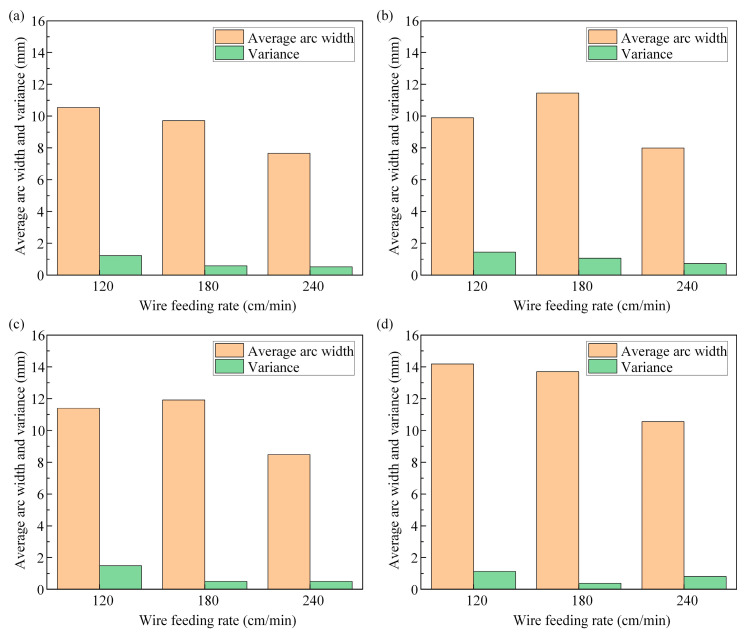
Histogram of the influence of welding speed changes on the average and variance of arc width: (**a**) wire feeding speed 5.5 m/min; (**b**) wire feeding speed 6.0 m/min; (**c**) wire feeding speed 6.5 m/min; (**d**) wire feeding speed 7.0 m/min.

Figure 9 shows the influence of wire feeding speed changes on the average and variance of arc width. The method of taking this average value was to measure the average arc width of the eight layers of additive under each process parameter. The variance was calculated to evaluate the difference between the arc width of each layer and the average arc width of the eight layers under this parameter, and the stability of the arc under this parameter was judged through data volatility. If the variance value of the arc width was small, this meant that the data volatility was small, and the arc had good stability during the multi-layer printing process. However, the variance value of the arc width was relatively large, which meant that the data volatility was high, indicating that the stability of the arc was poor during the multi-layer printing process.

In summary, to obtain thin-walled structures, when the welding speed was maintained at 240 cm/min and the wire feeding speed was 6.5 m/min, the variance value of the arc width was the lowest. This meant that during the process of printing eight layers of arc width under this parameter, the fusion amount of each layer matched the lifting amount of each layer of the welding gun, and the arc length and width basically did not change. Therefore, the stability of the printed arc was the highest under this process parameter.

## 4. Conclusions

(1) The process parameters had a critical impact on the quality of the coating layer formation. At a wire feed speed of 6.5 m/min, the resulting coating exhibited a smooth and glossy surface finish, with excellent weld continuity and wave uniformity.
(2) In the current process window, the short circuit phenomenon of the arc triggered periodic changes in current and voltage signals, that is, the arc formed, and the wire was fed forward until a droplet short circuit was formed. At this moment, the wire feeding speed was reversed, the wire was pulled back, and the current and voltage were almost zero. After the next open circuit was formed, the arc reignited, the welding wire was stopped and sent forward again, and the droplet transition began again. When the voltage suddenly dropped to 0 V and the current reached 300 A, this indicated that the welding wire was embedded in the molten pool or there was insufficient heat input, resulting in a lack of fusion and an increase in voids within the cladding layer.
(3) There was a negative correlation between droplet size and welding speed, where higher welding speeds resulted in reduced heat input, leading to smaller droplets. In addition, with the increase in the number of cladding layers, the distance between the welding gun and the substrate further reduced heat transfer, resulting in a decrease in droplet size.
(4) When the welding speed was maintained at 240 cm/min and the wire feeding speed was 6.5 m/min, the variance of the arc width was the smallest. Under these process parameters, the stability of the welding arc was the highest.

## Figures and Tables

**Table 1:** Chemical compositions of AA 2319 wire and AA 2219 substrate in wt.%.

Element	Si	Fe	Cu	Mn	Mg	Zn	Ti	Zr	V	Al
2319 wire	0.0420	0.0672	6.7381	0.2283	0.0066	0.0034	0.1132	0.1182	0.0845	Bal.
2219 substrate	0.0671	0.0823	4.1335	0.4663	0.0166	0.0559	0.0349	0.0003	0.0098	Bal.

**Table 2:** Summary of AA2319 WAAM process parameters.

Experimental Case	Current (*I*) (A)	Voltage (*U*) (V)	Wire Feeding Speed (*v_f_*) (m/min)	Welding Speed (*v_w_*) (cm/min)
1	90	10.7	5.5	**120**
2	90	10.7	5.5	**180**
3	90	10.7	5.5	**240**
4	120	13.4	6.0	**120**
5	120	13.4	6.0	**180**
6	120	13.4	6.0	**240**
7	131	14.6	6.5	**120**
8	131	14.6	6.5	**180**
9	131	14.6	6.5	**240**
10	140	15.0	7.0	**120**
11	140	15.0	7.0	**180**
12	140	15.0	7.0	240

## Data Availability

The original contributions presented in the study are included in the article, further inquiries can be directed to the corresponding authors.
